# Feasibility of using a mobile cardiotocograph device in an ambulance during maternal transport

**DOI:** 10.1371/journal.pone.0361118

**Published:** 2026-10-30

**Authors:** Shunsuke Tamaru, Natsuko Takano, Seung Chik Jwa, Hirokazu Ogawa, Takumi Kasumizawa, Atsushi Iwata, Seishi Shimizu, Tadashi Uesato, Jyunzo Hobo, Koji Samejima, Akikazu Nakamura, Kotaro Fukushima, Yoshiyasu Hirata, Tomoyuki Fujii, Isamu Ishiwata, Yoshimasa Kamei

**Affiliations:** 1 Department of Obstetrics and Gynecology, Saitama Medical University, Saitama, Japan; 2 Japan Association of Obstetricians and Gynecologists Telemedicine Proof-of-Concept Research Group, Tokyo, Japan; 3 Department of Obstetrics and Gynecology, Jichi Medical University, Tochigi, Japan; 4 Ogawa Maternity Clinic, Saitama, Japan; 5 Kasumizawa Sanfujinka, Saitama, Japan; 6 Iwata Ladies Clinic, Saitama, Japan; 7 Shimizu Hospital, Saitama, Japan; 8 Aiwa Hospital, Saitama, Japan; 9 Kikuchi Hospital, Saitama, Japan; 10 Samejima Bonding Clinic, Saitama, Japan; 11 Nakamura Maternity Clinic, Saitama, Japan; 12 Fukushima Clinic, Fukuoka, Japan; 13 Hirata Clinic, Saitama, Japan; 14 Department of Obstetrics and Gynecology, Sanno Hospital, Tokyo, Japan; 15 Ishiwata Obstetrics and Gynecology Hospital, Ibaraki, Japan; Saitama Medical Center: Saitama Ika Daigaku Sogo Iryo Center, JAPAN

## Abstract

Maternal transfer generally refers to the emergency transportation of a pregnant woman to a medical facility with more advanced capabilities when her condition, or that of the fetus, deteriorates or is expected to deteriorate, thereby ensuring the safety of both mother and fetus. This procedure is frequently performed in Japan, where childbirth at primary obstetric facilities is common. However, no reports have described attempts to remotely monitor maternal and fetal vital signs inside an ambulance during transport and use these data to support clinical care at a remote location. The current study aimed to assess the feasibility of a system that enables real-time remote monitoring (RM) using a small mobile cardiotocograph (CTG) in an ambulance during maternal transport from primary obstetric facilities to a higher-level perinatal medical center. This study included women with singleton pregnancies beyond 22 weeks of gestation who were transferred from nine private clinics to a university hospital because of medical complications. Using a small mobile CTG device, the CTG data were transmitted during maternal transfer to a university hospital. The obstetrician of the university hospital checked the data in real-time using tablets to select the appropriate treatment. A questionnaire survey was conducted on the medical staff of the primary facilities who accompanied the patients during maternal transport. An online survey was also administered to paramedics only after the recruitment period. The study period was from August 2021 to August 2023. In total, 68 pregnant women were enrolled in this study and analyzed. The median time from getting into the ambulance to arrival at the university hospital was 34.5 (range: 15.0–67.0) min. No monitoring sessions were discontinued because of connectivity issues. All 68 fetal heart rate records were legible, and the median proportion of the total legible period in each recording was 91.1% (range: 53.9%–99.8%). Twenty-three (33.8%) women delivered by the next day after the transportation. The questionnaire survey revealed high levels of medical staff satisfaction and paramedical acceptance of real-time CTG RM during maternal transfer. Real-time CTG RM during emergency maternal transport is feasible and useful for making a medical decision more precisely in tertiary perinatal medical centers. Further, the use of the system in an ambulance is also acceptable for paramedics.

## Introduction

Cardiotocography (CTG) is widely used in late pregnancy and labor management as a method of assessing fetal well-being, particularly for the early detection of fetal hypoxia. CTG continuously records fetal heart rate (FHR) changes and their association with uterine contractions. Hence, it is a useful indicator for examining fetal autonomic activity and oxygenation status [1].

In recent years, with the advancement of information and communication technology, performing real-time remote fetal monitoring using CTG has become feasible. This technology has several advantages. For example, it enables fetal monitoring at home and information sharing between local medical institutions and perinatal medical centers. Further, it allows continuous monitoring of fetal well-being without compromising the pregnant woman [2]. In particular, women with high-risk pregnancies, such as those with gestational hypertension, diabetic complications, and fetal growth restriction, require frequent monitoring. However, the high burden of hospital visits has been a clinical issue. Under these circumstances, CTG devices using Bluetooth, Wi-Fi, and 5G communications are gradually being used to automatically transmit real-time data from the pregnant woman’s home to the medical facility. This enables physicians to immediately identify signs of abnormality from a distance [3]. Moreover, in intrapartum monitoring, remote sharing of CTG data with perinatal medical centers at private obstetric clinics or hospitals enables the early detection of abnormal waveforms and appropriate transport decisions, thereby contributing to optimizing the timing of maternal transport and reducing delivery risks [4].

Recently, remote monitoring (RM) and telemedicine have rapidly developed in home and post-discharge patient care. However, the use of real-time RM in emergency medical services (EMS) has not yet become a commonplace [5]. Existing studies have theoretically proposed remote triage using wearable devices and artificial intelligence (AI). Nevertheless, there are no prospective studies that have validated a system that could share real-time biometric information with hospitals during actual EMS transport [6]. In the field of obstetrics, maternal transfer generally refers to the emergency transport of a pregnant woman to a medical facility with more advanced capabilities, ensuring the safety of both mother and fetus when the condition of either is deteriorating or expected to deteriorate. This procedure is frequently performed in Japan, where deliveries at primary obstetric facilities are common. However, to date, no studies have reported the remote monitoring of maternal and fetal vital signs inside an ambulance during transport and the use of these data to support clinical care at the receiving facility. Therefore, the current study aimed to assess the feasibility of a system that enables real-time RM using a small mobile CTG in an ambulance during maternal transport from private clinics to a perinatal medical center.

## Materials and methods

A prospective clinical study using a small mobile CTG (iCTG, Melody International Ltd., Kagawa, Japan) was performed. This study was approved by the institutional review board of Saitama Medical University Hospital (approval number: 2021−008; approval date: April 2021), and it was conducted as a project of the Information Technology Subcommittee of the Japanese Society of Obstetricians and Gynecologists. Written informed consent was obtained from all participants during the recruitment process prior to the initiation of data collection.

The participants were women with singleton pregnancies beyond 22 weeks of gestation who were maternally transferred to Saitama Medical University Hospital, which is the only regional perinatal medical center in the west area of Saitama from 9 nearby private obstetric clinics for obstetric complications or other reasons. Women who did not agree to participate in the study were excluded during recruitment. The iCTG was deployed at each clinic, and the iCTG was attached to the participants during maternal transport via ambulance to Saitama Medical University Hospital. Participants also wore pulse oximeters for continuous monitoring of oxygen saturation and maternal heart rate.

The medical staff of the clinics who accompanied the patients during maternal transfer checked the CTG data during the transport using a tablet, and the data were transmitted in a cloud server via the internet. Then, the obstetrician at the receiving facility (i.e., Saitama Medical University Hospital) confirmed the data in real-time on another tablet placed in the hospital obstetric ward for decision-making regarding the appropriate treatment. If transmission was temporarily interrupted, the accompanying healthcare professional checked the transducer placement and device connectivity. Data were stored locally during the interruption and automatically retransmitted once connectivity was restored. If transmission could not be restored, standard transport management based on maternal vital signs and clinical findings was continued. The duration and acquisition rate of legible CTG recordings during the maternal transfer were evaluated. The time interval from the maternal transfer to the delivery and the reason for transport in each case were also investigated. The travel distance by car from each clinic to Saitama Medical University Hospital was calculated using the functions of Google Map (https://www.google.com/maps).

In addition, a questionnaire survey was administered to the medical staff who accompanied the participants during the transport. The survey was conducted by providing ratings on a scale of 1–5 in each question (1 = strongly agree, 2 = agree, 3 = neutral, 4 = disagree, and 5 = strongly disagree). The questions asked were as follows:

1. Was it easy to use CTG monitoring during maternal transfer?
2. Was it secure to use CTG monitoring during maternal transfer?
3. Would you like to use CTG monitoring again during maternal transfer?

Moreover, an online survey using the Google form was performed on the paramedics in the study area after the recruitment period. The survey included questions listed below, and the results were rated on the same scale as the survey for the medical staff. The questions asked were as follows:

1. Is it safe to use CTG monitoring during maternal transfer?
2. Is it reassuring to use CTG monitoring during maternal transfer?
3. Is it worth to introduce the use of CTG monitoring during maternal transfer?
4. Is it burdensome to use CTG monitoring during maternal transfer?

The study period was from August 2021 to Augst 2023, and the annual number of deliveries at the participating research facilities during this period was approximately 8,000. The study variables were expressed as mean and standard deviation or median and range or numbers and percentage. All statistical data were analyzed using Statcel 2 for Windows (OMS, Tokyo, Japan).

## Results

In total, 68 pregnant women were enrolled in this study, and none of the women disagreed to participate in the study. Table 1 shows the characteristics of the participants.

**Table 1:** Clinical and social characteristics of the participants.

Number of participants	N = 68
Age (years)	31.9 ± 6.2
Gestational weeks at the time of transfer	33.1 ± 3.7
Parity	
0	37 (54.4%)
≥1	31 (45.6%)
BMI at time of transfer	
<18.5	1 (1.5%)
18.5–25.0	37 (54.4%)
25.0–30.0	19 (27.9%)
30.0–35.0	10 (14.7%)
≥35.0	1 (1.5%)
Reason for maternal transfer	
Preterm labor	32 (47.1%)
Preterm premature rupture of membrane	17 (25.0%)
Hypertensive disorder of pregnancy	11 (16.2%)
COVID-19 with labor	3 (4.4%)
Placental abruption	2 (2.9%)
Fetal growth restriction	1 (1.5%)
Low-lying placenta with abnormal bleeding	1 (1.5%)
Unbooked mother in labor	1 (1.5%)

Note: Data were presented as the mean ± SD or n (%).

The mean gestational age at time of maternal transfer was 33.1 ± 3.7 weeks, and the most frequent reason for maternal transfer was preterm labor (n = 32, 47.1%). Table 2 shows the travel distance from each clinic to Saitama Medical University Hospital. The median distance was 27.9 (range: 9.5–39.3) km.

**Table 2:** Travel distance from each of the nine participating clinics to Saitama Medical University Hospital.

Clinic No.	Travel Distance (km)
1	9.5
2	9.6
3	15.2
4	18.2
5	27.9
6	31.6
7	32.0
8	32.2
9	39.3

The median time from getting into the ambulance to the arrival at Saitama Medical University Hospital was 34.5 (range: 15.0–67.0) min. No monitoring sessions were discontinued due to connectivity issues. All 68 FHR records were legible, and the median percentage of the total legible period in each recording was 91.1% (range: 53.9%–99.8%). Fig 1 presents the distribution of the number and percentage of legible periods in each fetal heart rate measurement. According to the 2008 National Institute of Child Health and Human Development Workshop Report on the Electronic Fetal Monitoring of Three-Tier Fetal Heart Rate Interpretation System [7], the FHR patterns were classified as follows: Category I, n = 67 (98.5%); Category II, n = 1 (1.5%); and Category III, n = 0.

**Fig 1:**
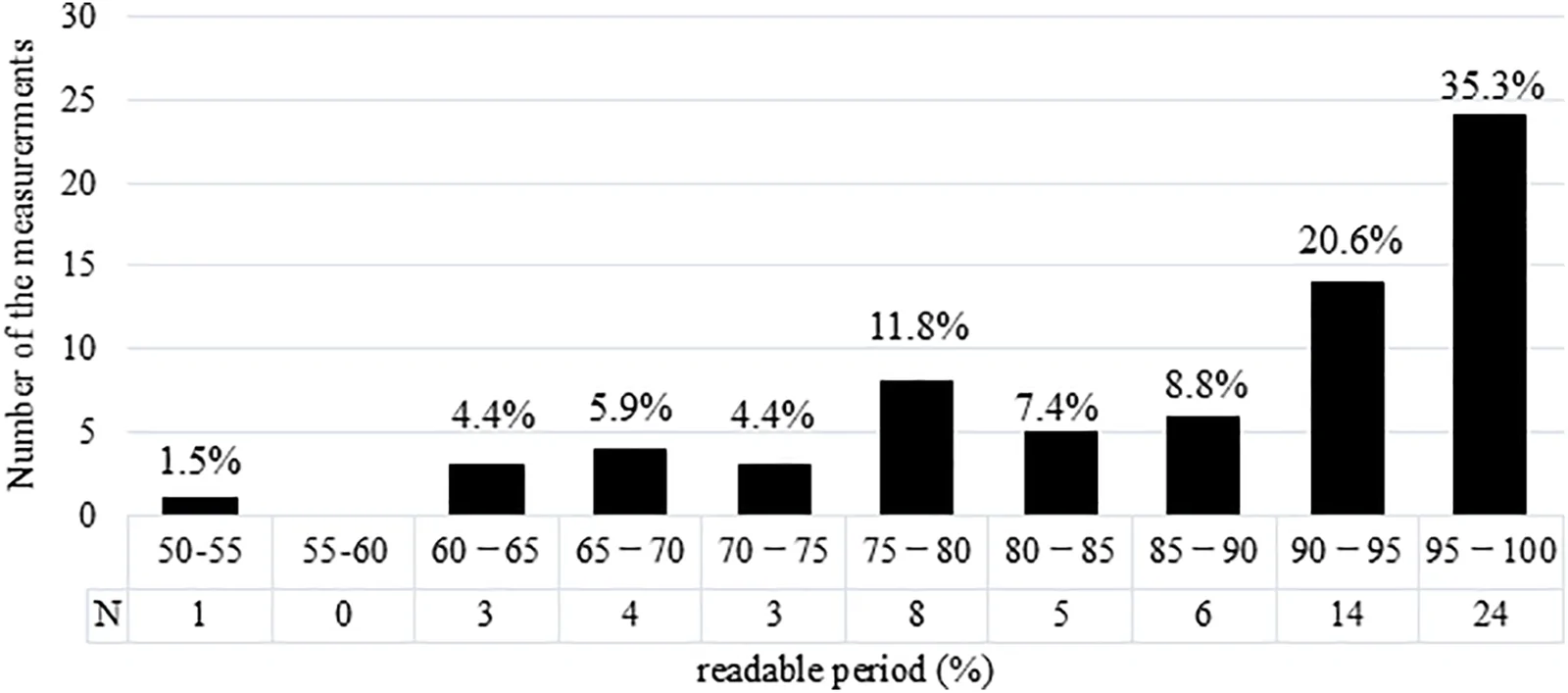
Distribution of the duration and percentage of legible periods in each of the 68 fetal heart rate recordings.

Fig 2a depicts the distribution of the time interval from maternal transfer to delivery. The number and the rate of delivery on the next day after transportation were 23 and 33.8%, respectively. Fig 2b shows the number of participants stratified by the reason for maternal transfer and the time interval from maternal transfer to delivery. Of the two cases initially diagnosed with placental abruption prior to maternal transport, one diagnosis was ruled out upon arrival at the tertiary care facility. In the remaining 67 cases, no discrepancy was observed between the pre-transport and post-transport diagnoses.

**Fig 2:**
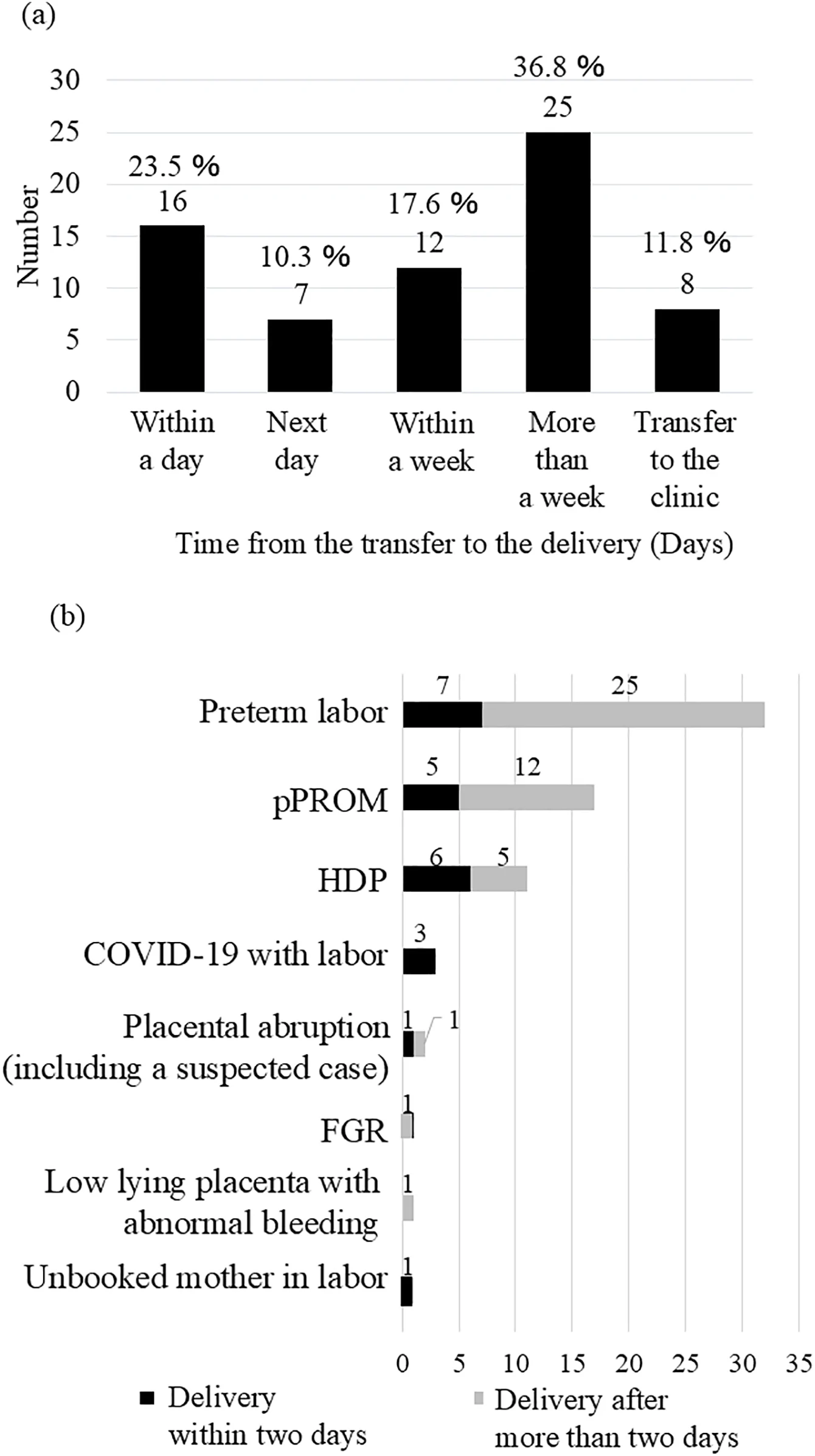
(a) Distri Distribution of the number and the percentage of the time from transfer to delivery in each participant. (b) Distribution of the number of the reasons for maternal transfer in each participant. The black bars show the number of women who delivered within 2 days after the transfer, and the gray bars depict the number of women who delivered after >2 days after the transfer. pPROM: preterm premature rupture of membranes, HDP: hypertensive disorders of pregnancy, FGR: fetal growth restriction.

There were no cases of childbirth during the transfer, perinatal death, or maternal death. One case involved a patient at 33 weeks and 1 day of gestation who presented to the clinic with chief complaints of abdominal pain and genital bleeding. The examination results raised suspicion of placental abruption; thus, the patient was transferred to another facility. The travel distance from the clinic to Saitama Medical University Hospital was 39.3 km, and the time from getting into the ambulance to arrival at the university hospital was 51.0 min. Fig 3 shows the CTG recording obtained during maternal transfer, confirming fetal well-being and frequent uterine contractions occurring every 2 min. Upon arrival at the hospital, placental abruption was confirmed during the initial evaluation, and an emergency cesarean section was immediately performed. Although the baby was admitted to the neonatal intensive care unit, both the mother and baby were discharged from the hospital without complications.

**Fig 3:**
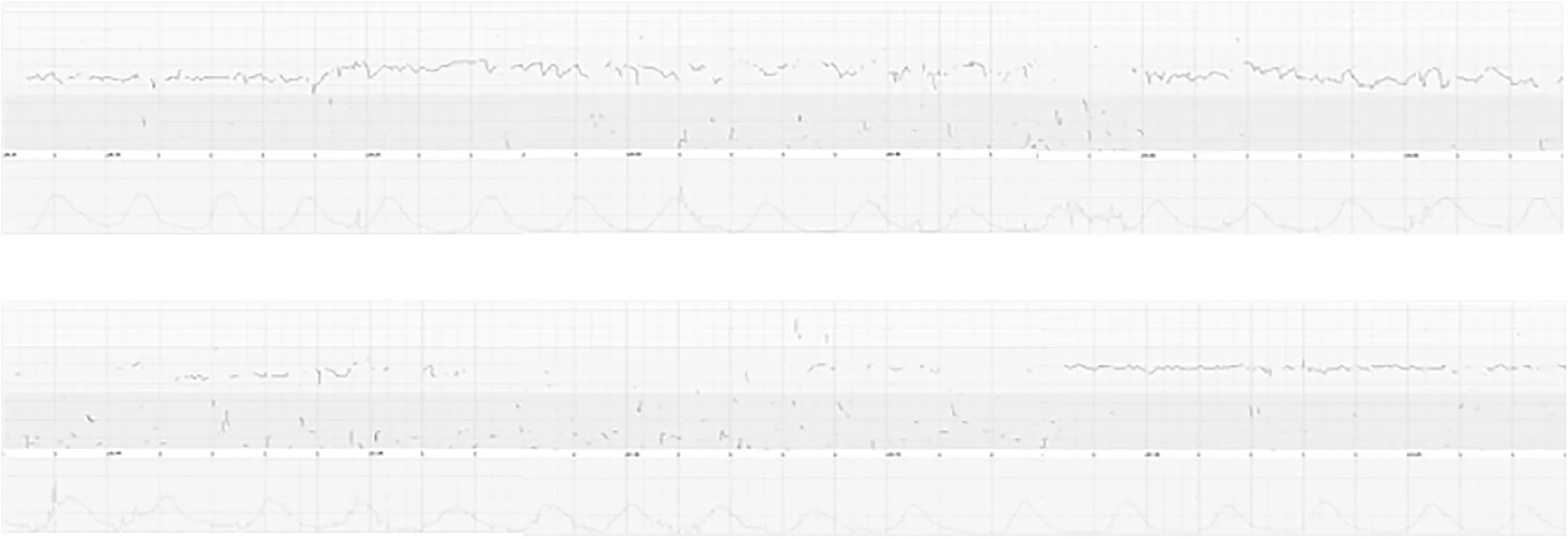
Cardiotocography recording obtained during maternal transfer in a case of placental abruption.

Of the 68 medical staff who answered the questionnaire survey, 11 (16.2%) were obstetricians, 30 (44.1%) midwives, and 27 (39.7%) nurses. Table 3 shows the scores for each question, and >80% of the medical staff responded positively in all three questions.

**Table 3:** Questionnaire survey performed on the 68 medical staff who accompanied the patients during maternal transfer.

Score and definition of each question	1 Strongly agree	2 Agree	3 Neutral	4 Disagree	5 Strongly disagree
Number of participants (%) Q1	29 (42.6)	26 (38.2)	10 (14.7)	3 (4.4)	0 (0)
Q2	58 (85.3)	8 (11.8)	2 (2.9)	0 (0)	0 (0)
Q3	58 (85.3)	8 (11.8)	2 (2.9)	0 (0)	0 (0)

Note: Data were expressed as n (%). Abbreviation: Q, question.

Q1. Was CTG monitoring during maternal transfer easy to use?

Q2. Did you feel that CTG monitoring during maternal transfer could be used safely and securely?

Q3. Would you like to use CTG monitoring again during maternal transfer?

For the questionnaire survey performed on the paramedics, 85 people answered. The years of job experience of the respondents were <1 year in 1 (1.2%) member, 1–5 years in 14 (16.5%), 5–10 years in 21 (24.7%), 10–15 years in 23 (27.1%), and >15 years in 26 (30.6%), respectively. Table 4 presents the scores for each question.

**Table 4:** Questionnaire survey conducted on 85 paramedics in the study area.

Score and definition of each question	1 Strongly agree	2 Agree	3 Neutral	4 Disagree	5 Strongly disagree
Number of participants (%) Q1	24 (28.2)	46 (54.1)	12 (14.1)	3 (3.5)	0 (0)
Q2	27 (31.8)	45 (52.9)	12 (14.1)	1 (1.2)	0 (0)
Q3	21 (24.7)	40 (47.1)	22 (25.9)	2 (2.4)	0 (0)
Q4	9 (10.6)	27 (31.8)	25 (29.4)	20 (23.5)	4 (4.7)

Note: Data were expressed as n (%). Abbreviation: Q, question.

Q1. Is it safe to use CTG monitoring during maternal transfer?

Q2. Is the use of CTG monitoring during maternal transfer reassuring?

Q3. Is it worthwhile to introduce CTG monitoring during maternal transfer?

Q4. Is the use of CTG monitoring during maternal transfer burdensome?

## Discussion

### Summary of main findings

This study showed that real-time CTG RM during emergency maternal transfer was safe and not difficult to use according to the medical staff who accompanied during the transport even though they needed to use it in a moving ambulance. Moreover, the real-time CTG RM during emergency maternal transfer was also acceptable for the paramedics in an ambulance. The quality of the CTG data in ambulances was sufficient for the physicians of the perinatal medical center to make a medical decision before patient arrival.

### Comparison with existing literature

The application of telemedicine and RM, including home monitoring and post-discharge monitoring, is rapidly increasing in the healthcare field [8]. However, there are only a few reports on the introduction of EMS that include the real-time RM of patients’ vital signs during emergency transport for remote medical teams and receiving hospitals [9]. Russi et al. have conducted a study showing the successful transmission and reception of real-time physiological summary data to a hospital base station during emergency patient transport via an ambulance or a helicopter using low-energy Bluetooth wireless wearable devices connected to Iridium satellite communications [5]. They successfully validated RM during transport in rural areas with poor communication environments for the transmission and reception of electrocardiograms and photoplethysmography waveforms. Based on these findings, continuous vital sign monitoring during the transport of patients with critically illness could enhance coordination with receiving medical facilities and potentially allow for changes in treatment plans prior to arrival if required. Although this report differs from our study as it focuses on monitoring during ambulance transport and does not include helicopter transport, it first showed the feasibility of the RM of FHR and maternal uterine contractions during emergency transport, thereby making it highly novel.

Regarding the RM of FHR and contraction patterns, a previous study evaluated the feasibility of wearing CTG at home in 101 patients with low-risk singleton pregnancies at ≥34 weeks of gestation [2]. This report indicated that the median percentage of the total CTG wear time during which evaluable CTG waveforms were obtained was 98.9%. According to this finding, pregnant women can obtain CTG data with a high probability even at home [2]. From the perspective of pregnant women, this study also evaluated satisfaction with self-monitoring of FHR using a mobile CTG device. Most participants reported high levels of satisfaction regarding learnability, ease of use, absence of anxiety, and expectations for the future use of self-guided FHR monitoring [2]. Further, Pilarczyk et al. reported that accurate data can be obtained with a high probability using CTG that is self-applied by pregnant women at home [10]. In our study, CTG was applied by midwives, nurses, or physicians who accompany the mothers during transport, which is expected to yield CTG waveforms at a higher rate than when applied by the pregnant woman herself. However, the median percentage of time during which evaluable CTG waveforms were obtained was 91.1%, which is lower than that in previous reports. This was assumed to be attributed to the difficulty of maintaining transducer position on the maternal abdominal wall within the moving ambulance environment and the smaller fetal size compared with previous studies, likely due to earlier gestational age. Nevertheless, since none of the CTG waveforms were not completely unreadable, the practice was considered feasible.

Several studies have also examined perinatal outcomes with self-applied CTG outside of hospitals. Suematsu et al. have reported that in 17 women with singleton pregnancies who were diagnosed with fetal growth restriction, the outpatient management of pregnant women was safely achieved via home CTG monitoring through real-time data review performed by hospital physicians [3]. Moreover, there are other reports about patient management, including home CTG self-application, not only for women with low-risk pregnancies but also for those with obstetric complications [11,12]. In these studies, no increase in the incidence of serious adverse events in either the mother or the newborn was observed when comparing home management with inpatient management. Consequently, home CTG self-application was considered a valid management method. Our study is the only report on out-of-hospital CTG monitoring, specifically during transportation via an ambulance. As this research presents findings under conditions distinct from known reports showing data from self-applied CTG, it is considered highly novel.

### Strengths and limitations

Our study is not a comparative trial between groups. Therefore, it does not compare maternal and fetal perinatal outcomes between cases where CTG was applied in the ambulance and those where it was not during maternal transport. Consequently, this study did not validate whether maternal and fetal perinatal outcomes improve compared with conventional maternal transport methods. Additionally, cost-effectiveness was not examined. However, the CTG information obtained in real-time from the ambulance is supplementary to all information obtained prior to patient arrival during conventional maternal transport. We believe that the increased amount of information obtained in the ambulance does not constitute a disadvantage in management compared with conventional methods that do not perform CTG monitoring during transport.

The participants in this study differed in gestational age, body weight, and indications for transport. Therefore, it is not feasible to evaluate the usefulness of this system according to specific gestational age, weight, or indication. In cases of extremely early gestational age, obtaining FHR data may be difficult due to the small fetal size. Even if monitoring is possible, interpretation of fetal well-being may be challenging due to immaturity of the fetal central nervous system. Moreover, in cases of extreme maternal obesity, the distance between the fetal heart and the transducer may be greater, potentially making it difficult to obtain reliable FHR data. In this study, a detailed evaluation of whether real-time FHR monitoring is feasible in cases of severe obesity was not conducted; therefore, this remains unknown. In addition, this study was specifically designed to assess the feasibility of implementing real-time CTG remote monitoring using a small mobile CTG device. Evaluation of its clinical usefulness remains a future task and is a limitation of this study.

Regarding the mobile CTG device, data transmission is possible in environments with internet or Wi-Fi access. In Japan, mobile network coverage for internet access is nearly 100% in populated areas. Communication generally does not drop out, at least in environments such as roads where ambulances operate. However, in areas with poor connectivity or in cases of equipment malfunction, use of this system becomes challenging, which is a limitation of the system.

This study was conducted between nine facilities in relatively densely populated areas of Saitama Prefecture with reliable cellular coverage and a single perinatal center. The maximum transport distance and duration were 39.3 km and 67 min, respectively, and most FHR tracings were classified as Category I. Therefore, our findings may not be generalizable to rural or mountainous areas with limited connectivity, longer transports, multiple receiving centers, or cases involving severe fetal compromise. Further multicenter studies in diverse geographic and communication settings should assess the frequency and duration of connectivity interruptions.

As for the comparison between remote CTG monitoring during maternal transport and having a physician accompany the ambulance to monitor CTG while communicating with hospital physicians via telecommunication, the situation in Japan is as follows: due to the limited number of physicians at each private obstetric clinic, it is generally difficult for physicians to accompany ambulances. Instead, midwives or nurses typically accompany patients. Therefore, when sufficient physicians are available, management via telecommunication may be possible. However, even in that case, physicians at the receiving hospital would not be able to directly view CTG data. We therefore believe that a system similar to that implemented in this study would be useful in areas with a shortage of physicians. The appropriate approach will vary depending on local circumstances.

Further, questionnaire results from accompanying medical personnel and paramedics clearly indicate that performing CTG monitoring in the ambulance plays a significant role in ensuring a safer and more secure maternal transport, yielding highly significant results. However, the present study did not examine the extent to which participants accepted the use of CTG monitors in ambulances. This issue warrants further investigation.

### Future developments

The next step toward the practical implementation of our system may ultimately involve developing a machine learning algorithm. This algorithm would detect subtle changes in transmitted FHR and uterine contraction data, thereby alerting the receiving team to physiological changes. If such a system can be developed, with adequate physiological and patient background data, AI could potentially support the transport team and the receiving team by predicting the risk of clinical deterioration, as is currently being explored by other researchers [13]. Our study did not examine pregnancy outcomes and cost-benefit considerations. Nevertheless, we believe that an intervention study should be conducted to determine whether performing CTG RM improves pregnancy outcomes and whether a healthcare economic analysis should be conducted to examine the actual implementation of our system.

## Conclusions

Real-time CTG RM using a small mobile cardiotocograph device during emergency maternal transport is feasible and useful in making more precise decisions among physicians in higher-level perinatal medical centers. Further, the use of the system in an ambulance is acceptable among medical staff and paramedics. Nevertheless, in the future, further multicenter research should be performed to assess the impact of real-time CTG RM during emergency maternal transportation on the mothers and their babies.

## Supporting information

S1 Data Anonymized minimal dataset underlying the findings of this study. The spreadsheet contains the data underlying the reported summary statistics, questionnaire results, and figures.
(XLSX)
