# Supplementary material for: Feasibility of using a mobile cardiotocograph device in an ambulance during maternal transport
Source: PLoS One. 2026 Oct 30;21(10):e0361118. doi: 10.1371/journal.pone.0361118 (PMC13722184; doi:10.1371/journal.pone.0361118)
Supplement: Supplementary file 1 [file pone.0361118.s003.docx]

**Point-by-point responses to the reviewer:**

Journal Requirements:

When submitting your revision, we need you to address these additional requirements.

1.Please ensure that your manuscript meets PLOS ONE's style requirements, including those for file naming. The PLOS ONE style templates can be found at

https://journals.plos.org/plosone/s/file?id=wjVg/PLOSOne_formatting_sample_main_body.pdf and

<https://journals.plos.org/plosone/s/file?id=ba62/PLOSOne_formatting_sample_title_authors_affiliations.pdf>

**Response:**

Thank you for this comment. We have reconfirmed that the manuscript meets PLOS ONE's style requirements, including the file naming convention.

2. Thank you for stating the following in the Competing Interests/Financial Disclosure * (delete as necessary) section:

“I have read the journal's policy and the authors of this manuscript have the following competing interests. Melody International, Ltd. (Takamatsu, Japan), loaned the iCTG devices to the study group free of charge.”

We note that you received funding from a commercial source: “Melody International, Ltd.”

Please provide an amended Competing Interests Statement that explicitly states this commercial funder, along with any other relevant declarations relating to employment, consultancy, patents, products in development, marketed products, etc.

Within this Competing Interests Statement, please confirm that this does not alter your adherence to all PLOS ONE policies on sharing data and materials by including the following statement: "This does not alter our adherence to PLOS ONE policies on sharing data and materials.” (as detailed online in our guide for authors http://journals.plos.org/plosone/s/competing-interests).  If there are restrictions on sharing of data and/or materials, please state these. Please note that we cannot proceed with consideration of your article until this information has been declared.

Please include your amended Competing Interests Statement within your cover letter. We will change the online submission form on your behalf.

**Response:**

Thank you for this comment. I provided an amended Competing Interests Statement about Melody International, Ltd. in our cover letter for the revised manuscript. The amended Competing Interests Statement is provided below.

I have read the journal's policy and the authors of this manuscript have the following competing interests. Melody International, Ltd. (Takamatsu, Japan), loaned the iCTG devices to the study group free of charge. In addition to the above, there are no financial competing interests or non-financial competing interests for any of the authors related to Melody International, Ltd. Melody International, Ltd. had no role in the conception, preparation, or decision to publish this article. This does not alter our adherence to PLOS ONE policies on sharing data and materials.

3. In the online submission form, you indicated that your data is available only on request from a third party. Please note that your Data Availability Statement is currently missing [the name of the third party contact or institution / contact details for the third party, such as an email address or a link to where data requests can be made]. Please update your statement with the missing information.

**Response:**

Thank you for this comment. According to the comment, we amended the Data Availability Statement as below.

Data cannot be shared publicly because the data used for this study include personal information and are ethically restricted by the Institutional Review Board of Saitama Medical University Hospital. However, data are available from the Institutional Review Board of Saitama Medical University Hospital (contact via hirb@saitama-med.ac.jp) for researchers who meet the criteria for access to confidential data on reasonable request.

4. One of the noted authors is a group or consortium “Japan Association of Obstetricians and Gynecologists Telemedicine Proof-of-Concept Research Group” In addition to naming the author group, please list the individual authors and affiliations within this group in the acknowledgments section of your manuscript. Please also indicate clearly a lead author for this group along with a contact email address.

**Response:**

Thank you for this comment. To be precise, some authors of the paper are members of the Japan Association of Obstetricians and Gynecologists Telemedicine Proof-of-Concept Research Group, and we have revised the authors' affiliations to reflect this fact.

5. Please include your full ethics statement in the ‘Methods’ section of your manuscript file. In your statement, please include the full name of the IRB or ethics committee who approved or waived your study, as well as whether or not you obtained informed written or verbal consent. If consent was waived for your study, please include this information in your statement as well.

**Response:**

Thank you for this comment. Regarding ethical procedures, the entries in the Materials and Methods section have been revised as follows.

This study was approved by the institutional review board of Saitama Medical University Hospital (approval number: 2021-008; approval date: April 2021), and it was conducted as a project of the Information Technology Subcommittee of the Japanese Society of Obstetricians and Gynecologists. Prior to initiating data collection, written informed consent was obtained from all participants during the recruitment process (lines 104-109).

6. Please upload a copy of Figure 1, 2, 3 and 4, to which you refer in your text on page 10, 11 and 12. If the figure is no longer to be included as part of the submission please remove all reference to it within the text.

**Response:**

Thank you for this comment. Following the comments, we have inserted each figure and figure legend into the main body of the manuscript.

7. We note that Figure 1 in your submission contain map images which may be copyrighted. All PLOS content is published under the Creative Commons Attribution License (CC BY 4.0), which means that the manuscript, images, and Supporting Information files will be freely available online, and any third party is permitted to access, download, copy, distribute, and use these materials in any way, even commercially, with proper attribution. For these reasons, we cannot publish previously copyrighted maps or satellite images created using proprietary data, such as Google software (Google Maps, Street View, and Earth). For more information, see our copyright guidelines: http://journals.plos.org/plosone/s/licenses-and-copyright.

We require you to either (1) present written permission from the copyright holder to publish these figures specifically under the CC BY 4.0 license, or (2) remove the figures from your submission:

1. You may seek permission from the original copyright holder of Figure 1 to publish the content specifically under the CC BY 4.0 license.

We recommend that you contact the original copyright holder with the Content Permission Form (http://journals.plos.org/plosone/s/file?id=7c09/content-permission-form.pdf) and the following text:

“I request permission for the open-access journal PLOS ONE to publish XXX under the Creative Commons Attribution License (CCAL) CC BY 4.0 (http://creativecommons.org/licenses/by/4.0/). Please be aware that this license allows unrestricted use and distribution, even commercially, by third parties. Please reply and provide explicit written permission to publish XXX under a CC BY license and complete the attached form.”

Please upload the completed Content Permission Form or other proof of granted permissions as an "Other" file with your submission.

In the figure caption of the copyrighted figure, please include the following text: “Reprinted from [ref] under a CC BY license, with permission from [name of publisher], original copyright [original copyright year].”

2. If you are unable to obtain permission from the original copyright holder to publish these figures under the CC BY 4.0 license or if the copyright holder’s requirements are incompatible with the CC BY 4.0 license, please either i) remove the figure or ii) supply a replacement figure that complies with the CC BY 4.0 license. Please check copyright information on all replacement figures and update the figure caption with source information. If applicable, please specify in the figure caption text when a figure is similar but not identical to the original image and is therefore for illustrative purposes only.

The following resources for replacing copyrighted map figures may be helpful:

USGS National Map Viewer (public domain): http://viewer.nationalmap.gov/viewer/

The Gateway to Astronaut Photography of Earth (public domain): http://eol.jsc.nasa.gov/sseop/clickmap/

Maps at the CIA (public domain): https://www.cia.gov/library/publications/the-world-factbook/index.html and https://www.cia.gov/library/publications/cia-maps-publications/index.html

NASA Earth Observatory (public domain): http://earthobservatory.nasa.gov/

Landsat: http://landsat.visibleearth.nasa.gov/

USGS EROS (Earth Resources Observatory and Science (EROS) Center) (public domain): http://eros.usgs.gov/#

Natural Earth (public domain): <http://www.naturalearthdata.com/>

**Response:**

Thank you for this comment. According to the comment, we removed the map of the Figure 1 from our submission. We have also changed the numbers of each figure and table in accordance with this revision.

8. We notice that your supplementary figures are uploaded with the file type 'Figure'. Please amend the file type to 'Supporting Information'. Please ensure that each Supporting Information file has a legend listed in the manuscript after the references list.

**Response:**

Thank you for this comment. This paper does not contain supplementary figures. What was uploaded during the initial submission was not supplementary figures, but figures. In the revised manuscript, the figures have been inserted into the main text and also uploaded as a separate file.

9. If the reviewer comments include a recommendation to cite specific previously published works, please review and evaluate these publications to determine whether they are relevant and should be cited. There is no requirement to cite these works unless the editor has indicated otherwise. 
**Response:**

Thank you for this comment. We carefully checked the comments from the reviewers, but there were no instructions to search for or cite specific additional articles.

[Note: HTML markup is below. Please do not edit.]

Reviewer's Responses to Questions

**Comments to the Author**

1. Is the manuscript technically sound, and do the data support the conclusions?

The manuscript must describe a technically sound piece of scientific research with data that supports the conclusions. Experiments must have been conducted rigorously, with appropriate controls, replication, and sample sizes. The conclusions must be drawn appropriately based on the data presented.

Reviewer #1: Partly

Reviewer #2: Yes

2. Has the statistical analysis been performed appropriately and rigorously?

Reviewer #1: N/A

Reviewer #2: I Don't Know

3. Have the authors made all data underlying the findings in their manuscript fully available?

The [PLOS Data policy](http://www.plosone.org/static/policies.action#sharing) requires authors to make all data underlying the findings described in their manuscript fully available without restriction, with rare exception (please refer to the Data Availability Statement in the manuscript PDF file). The data should be provided as part of the manuscript or its supporting information, or deposited to a public repository. For example, in addition to summary statistics, the data points behind means, medians and variance measures should be available. If there are restrictions on publicly sharing data—e.g. participant privacy or use of data from a third party—those must be specified.

Reviewer #1: No

Reviewer #2: No

4. Is the manuscript presented in an intelligible fashion and written in standard English?

PLOS ONE does not copyedit accepted manuscripts, so the language in submitted articles must be clear, correct, and unambiguous. Any typographical or grammatical errors should be corrected at revision, so please note any specific errors here.

Reviewer #1: Yes

Reviewer #2: Yes

5. Review Comments to the Author

Please use the space provided to explain your answers to the questions above. You may also include additional comments for the author, including concerns about dual publication, research ethics, or publication ethics. (Please upload your review as an attachment if it exceeds 20,000 characters)

Reviewer #1: Thank you for the opportunity to review this paper.

1. I believe that the key of this manuscript is "What is new". I understood the usability of mobile CTG RM during the maternal transport has not been reported yet according to this manuscript. If so, I will find this manuscript sounds novel and would like authors to emphasize this point. I did not get "what is known" and "what is unknown" regarding this topic in the Abstract and Introduction, especially focusing on the obstetric insights. I would recommend that authors summarize them in the Abstract and the Introduction.

**Response:**

Thank you very much for your valuable comments. Following the reviewer's suggestions, we have added the recommended content to the Abstract and Introduction sections (lines 33-39, 91-98).

2. I would question if the authors can say "mobile CTG RM during emergency maternal transport is feasible" without the discussion about the cost of introduction of this system (line 50). Similarly, I would say it is questionable whether authors can say "it is at least not inferior to previous management methods" (line 251), because there is no comparison between them. I did not see the data regarding the cost and I am not sure if this can be used at very rural places and on the helicopter. I would like to get the information about what we can do if the system is broken. Moreover, I would like to read the discussion about CTG RM can be better for the management than telecommunication with the attending physician who can directly observe CTG during the maternal transport.
**Response:**

Thank you very much for your valuable comments. Unfortunately, this study did not examine cost-effectiveness. Therefore, we have added a discussion of this point as a limitation in the discussion section (lines 298-299).

Regarding the comment that “it is questionable whether authors can say "it is at least not inferior to previous management methods", because there is no comparison between them,” we have revised the relevant passage as follows.

“We believe that the increased amount of information obtained in the ambulance does not constitute a management disadvantage compared to conventional management methods that do not perform CTG monitoring in the ambulance” (lines 301-304).

Regarding this device, data transmission is possible in environments with internet or Wi-Fi access. In Japan, mobile phone network coverage for internet access is nearly 100% established in populated areas. Communication generally does not drop out, at least in environments like roads where ambulances travel. Furthermore, although not reported in academic papers, communication environments are also established in Japan in settings like helicopter-mediated patient transporting system from remote islands to the main island. Mobile CTG devices are used in clinical practice in some regions. However, in areas with poor communication environments or in cases of equipment malfunction, using this system becomes difficult and is considered a limitation of the system. Additionally, I have added a note to the “Strengths and Limitations” section under the Discussion heading regarding this matter (lines 320-326).

Regarding the comparison between remote monitoring of CTG during maternal transport and having a physician accompany the ambulance to monitor CTG while communicating with hospital physicians via telecommunication, the situation in Japan is as follows: Due to the limited number of Physicians on staff at each private obstetric clinic, it is generally difficult for physicians themselves to accompany ambulances. Instead, midwives or nurses typically accompany the patient. Therefore, if there are sufficient physicians, it is possible to opt for management via telecommunication. However, even in that case, the constraint remains that physicians at the receiving hospital could not directly view the CTG data. Therefore, we believe that a system like the one implemented in this study would be useful in areas with a shortage of physicians. Furthermore, we consider that the appropriate method will vary depending on local circumstances. Additionally, I have added a note to the “Strengths and Limitations” section under the Discussion heading regarding this matter (lines 327-338).

3. Speaking of Table 1, I was wondering if this system is useful for pregnant women <28 weeks of gestation. I was also wondering if this system is hard to attach to the pregnant women whose BMI >35 kg/m2. What is possible preterm labor? Is this different from preterm labor? I was wondering in how many cases authors observed abnormal fetal heart rate patterns. I was also wondering if the diagnosis before transport, during the transport, and after the transport are matched. I believe these parameters are important to discuss the feasibility of this device.
**Response:**

Thank you very much for your valuable comments.

In cases where gestational age is 28 weeks or earlier, it is considered that obtaining fetal heart rate data may be difficult due to the small size of the fetus. Even if monitoring is possible, evaluating fetal well-being based on the obtained waveforms may be challenging due to fetal immaturity of the central nervous system. Additionally, in cases where the mother is extremely obese, the distance between the fetal heart and the device transducer may increase, potentially making it difficult to acquire fetal heartbeat data. In this study, one case had a BMI of 36.5 at the time of maternal transport, but real-time data monitoring was possible for that case. However, in this study, detailed examination of whether real-time monitoring is feasible in cases of severe obesity has not been conducted, so it remains unknown. Each of these points is considered a limitation of this study. I have added these limitations to the Discussion section (lines 306-319).

The term “possible preterm labor” in the Table 1 was a simple error, so it has been corrected to “preterm labor.”

According to the 2008 National Institute of Child Health and Human Development Workshop Report on Electronic Fetal Monitoring of Three-Tier Fetal Heart Rate Interpretation System, the FHR patterns for each case are classified as follows: Category Ⅰ in 67 cases (98.5%), Category Ⅱ in 1 case (1.5%), and no cases in Category Ⅲ. One of the two cases diagnosed with placental abruption prior to maternal transport was subsequently ruled out upon arrival at the tertiary care facility. In the other 67 cases, there was no discrepancy between the pre- and post-transport diagnoses. Additional information regarding these research findings has been added to the main text (lines 170-174, 182-185).

Additionally, in accordance with this revision, Reference 7 has been added, and the numbers of the other references have been changed.

4. I would like to ask if ununified reasons for maternal transport and gestational ages in this analysis can be the potential limitations. I would say mobile CTG RM may not be required during the stable maternal transport like threatened preterm births just diagnosed with short cervix.
**Response:**

Thank you very much for your valuable comments. In Japan, private obstetric clinics handling deliveries do not have pediatricians on staff, and it is common for only midwives and obstetricians to attend births there. Therefore, maternal transfers are performed when the newborn may require some form of treatment or when the mother may need multidisciplinary care. Pregnant women diagnosed solely with cervical shortening and at risk for preterm labor are generally not candidates for maternal transfer and continue treatment at private obstetric clinics.

However, as the reviewers pointed out, the participants in this study varied in gestational age and maternal indications for transport. Therefore, it is not feasible to evaluate the usefulness of this system for each specific gestational age or maternal indication. This study was designed specifically to assess the feasibility of implementing this system. Evaluating its usefulness remains a future task and is considered a limitation of this study. Additionally, we have added the above points to the “Limitations” section of the Discussion (lines 306-319).

Minor comments:
I was wondering how long it would take to attach this system to the patient and it would get the signals (lines 98-100).
**Response:**

Thank you for this comment. The time required to attach this device is generally the same as for non-mobile CTG devices typically used in hospitals. Additionally, waveform detection is possible almost simultaneously with CTG attachment. In environments connected to the internet or Wi-Fi, remote waveform detection is possible almost simultaneously (within seconds) with CTG attachment.

I would recommend that authors include URL of Google Map (line 109).
**Response:**

Thank you for this comment. As requested in the comment, I have included the Google Maps URL within the relevant section of the text (line 128).

I was curious if maternal heart rate can be also monitored using this device (line 224).
**Response:**

Thank you for this comment. The equipment used in this study does not measure maternal heart rate. However, in Japanese ambulances, patients are always fitted with an oxygen saturation monitor, which inevitably monitors the mother's heart rate during transport. Therefore, accompanying medical personnel can easily distinguish between fetal and maternal heart rates. If it is determined that the fetal heart rate is not being measured, the position of the CTG monitor can be adjusted within the ambulance. We have added the following to the relevant section in the main text: All subjects wore oxygen saturation monitors, and maternal heart rate was also monitored (lines 117-118).

The footnotes are essential to indicate what Q1, Q2, Q3, and Q4 are asking in Table 2 and 3.

**Response:**

Thank you for this comment. As requested in the comment, I have included the content of each question within the footnotes of the Tables.

Reviewer #2: I am astonished that the authors consider it important to ask the paramedics their opinion of the acceptability of using CTGs during transport but make no mention at all regarding the views of the women themselves. I would only recommend publication if data concerning the acceptability to women is included. Neglecting to include the views of the women displays a lack of patient-centered care that is not appropriate. If this data is available, please include it. If it was not obtained then I cannot recommend publication, regardless of any other issues. I appreciate this is a proof of concept and from the point of view of technical feasibility the technique seems to have been demonstrated to work. However please revise if possible, taking the views of patients into account.

**Response:**

Thank you very much for your valuable comments. In this study, we did not conduct a questionnaire survey targeting pregnant women themselves, so we do not possess the data requested by the reviewer. However, in a separate study conducted previously, we used the same mobile CTG device as in this study to conduct research where pregnant women themselves wore the device at home, and healthcare professionals at the hospital reviewed the data (Tamaru S, et al. J Obstet Gynaecol Res. 2022;48: 385-392.). In that study, the participants also completed a questionnaire after delivery to evaluate their satisfaction with self-monitoring of FHR using the mobile CTG device. The majority of participants reported high levels of satisfaction with the learnability, ease of use, absence of anxiety and future expectations regarding self-guided FHR monitoring. Given this background, we were confident that pregnant women participating in this study, who were already concerned about their fetus's condition, would not doubt the benefits of being able to monitor the fetus during emergency transport. Therefore, we did not conduct a questionnaire survey of the pregnant women themselves in this study.

In light of the above, following the reviewer's comments, we have added descriptions from the perspective of pregnant women in the Discussion section (lines 257-261, 272-277).

6. PLOS authors have the option to publish the peer review history of their article ([what does this mean?](https://journals.plos.org/plosone/s/editorial-and-peer-review-process#loc-peer-review-history)). If published, this will include your full peer review and any attached files.

If you choose “no”, your identity will remain anonymous but your review may still be made public.

**Do you want your identity to be public for this peer review?** For information about this choice, including consent withdrawal, please see our [Privacy Policy](https://www.plos.org/privacy-policy).

Reviewer #1: No

Reviewer #2: No
