# Supplementary material for: Feasibility of using a mobile cardiotocograph device in an ambulance during maternal transport
Source: PLoS One. 2026 Oct 30;21(10):e0361118. doi: 10.1371/journal.pone.0361118 (PMC13722184; doi:10.1371/journal.pone.0361118)
Supplement: Supplementary file 2 [file pone.0361118.s004.docx]

**Point-by-point responses to the reviewer:**

Review Comments to the Author

Reviewer #1: I believe that all comments have been properly addressed. However, some English expressions still sound unnatural. I recommend that the authors have the manuscript reviewed by a native English speaker.

**Response:**

Thank you very much for your valuable comments. As with the submission of the first draft, we asked a native English-speaking scientist to review the manuscript and help us refine it for this third submission of the revised version. The revised sections are highlighted.

Reviewer #2: Thank you for your comment concerning the views of the patients. I appreciate that you did not survey the patients at the time of the survey, meaning that you have no data available concerning patient acceptability. You have justified this by stating that when patients used the monitoring device themselves at home they found it acceptable and this implies that therefore they would also find it acceptable during transport. However, I consider that these are two very different circumstances and acceptability in one cannot be taken to mean that use is necessarily acceptable in the other. I really cannot recommend this paper for publication unless it includes a reflection of the acceptability to the patients in the ciurcumstance of transport. I hope that you will reflect on this view going forward as the views of the patients should always be considered important.

**Response:**

Thank you very much for your valuable comments. As the reviewer pointed out, this study did not investigate the extent to which study participants accepted the use of fetal heart rate monitors during emergency transport. We have noted this as a limitation of the study in the Discussion section. In future research, we will carefully consider the point raised by the reviewer that our studies should be conducted from the perspective of the participants.
