# Supplementary material for: Feasibility of using a mobile cardiotocograph device in an ambulance during maternal transport
Source: PLoS One. 2026 Oct 30;21(10):e0361118. doi: 10.1371/journal.pone.0361118 (PMC13722184; doi:10.1371/journal.pone.0361118)
Supplement: Supplementary file 3 [file pone.0361118.s005.docx]

**Point-by-point responses to the reviewer:**

Review Comments to the Author

Reviewer #1:  I believe that all comments have been properly addressed and the current manuscript is written in standard English.

**Response:**

Thank you for confirming that all comments have been appropriately addressed and that the manuscript is written in standard English.

Reviewer #3: This study demonstrates clear feasibility and clinical value for mobile CTG during ambulance transport. Methodology is sound, questionnaire data strengthens acceptance findings, and the manuscript is well-written. I recommend minor revisions regarding generalizability and suggest clarifying how connectivity issues were managed during longer transports.

**Response:**

Thank you very much for your valuable comments. We added a statement to the Discussion addressing the generalizability of our findings in terms of the study setting, participating facilities, transport distance, connectivity, and FHR categories. We also clarified the procedures for managing connectivity issues, including during longer transports, in the Methods and noted in the Results that no connectivity-related problems occurred during the study.
