# Supplementary material for: Feasibility of using a mobile cardiotocograph device in an ambulance during maternal transport
Source: PLoS One. 2026 Oct 30;21(10):e0361118. doi: 10.1371/journal.pone.0361118 (PMC13722184; doi:10.1371/journal.pone.0361118)
Supplement: Supplementary file 4 [file pone.0361118.s006.docx]

Point by Point Responses to the Reviewers and Editorial Office

**Manuscript ID: PONE-D-25-57871R4**

**Title:** Feasibility of using a mobile cardiotocograph device in an ambulance during maternal transport

Dear Academic Editor and Reviewers,

Thank you for assessing our manuscript. We have addressed the reviewer comments and the subsequent Data Availability request as detailed below.

**Reviewer 1**

**Comment:** I confirmed that all of my comments had been appropriately addressed in the previous round of review. I believe that the manuscript has been substantially improved, particularly by the addition of a statement addressing the generalizability of the findings with respect to the study setting, participating facilities, transport distance, connectivity, and FHR categories.

**Response:** Thank you for your positive assessment and for confirming that our previous revisions addressed your comments.

**Reviewer 3**

**Comment:** The abstract provides a clear rationale for evaluating mobile cardiotocograph (CTG) devices during emergency maternal transport, but it lacks critical structure, results, and technical specifications regarding sample size and equipment, alongside a typographical error in the keywords ("moblie"). The text terminates prematurely without providing data transmission success rates or statistical outcomes, which are essential for a standard PLOS ONE submission.

**Response:** Thank you for your comments. We corrected “moblie” to “mobile” in the submission keywords. The abstract continues from page 3 onto page 4 of the manuscript PDF and reports the 68 participants, the mobile CTG device, quantitative results, and conclusions. We added: “No monitoring sessions were discontinued because of connectivity issues.” We did not calculate a separate data transmission success rate and have not described the absence of discontinuation as a 100% transmission success rate. We also corrected the transport-time median to 34.5 minutes (range, 15–67) after verification of the source record.

**Editorial office request concerning Data Availability**

Comment: If ethical or legal restrictions prevent public sharing of de-identified data, explain the restrictions and provide an institutional contact. Otherwise, make the minimal anonymized dataset publicly available in a repository or as Supporting Information, and update the Data Availability statement.

Response: Institutional permission for public release of the anonymized minimal dataset has been confirmed. The dataset is submitted as S1 Data in Supporting Information, and the Data Availability statement identifies this file. It includes individual values underlying summary statistics, questionnaire responses, and relevant figure data. Direct identifiers, age, dates, facility names, study codes, and free-text clinical notes have been excluded.
