# Supplementary material for: Feasibility of using a mobile cardiotocograph device in an ambulance during maternal transport
Source: PLoS One. 2026 Oct 30;21(10):e0361118. doi: 10.1371/journal.pone.0361118 (PMC13722184; doi:10.1371/journal.pone.0361118)
Supplement: Peer review history

# Peer review: Decision Letter 0

**DOI:** 10.1371/journal.pone.0361118.r001

**Authors:** Agnese Sbrollini

15 Feb 2026

Dear Dr. Tamaru,

Thank you for submitting your manuscript to PLOS ONE. After careful consideration, we feel that it has merit but does not fully meet PLOS ONE’s publication criteria as it currently stands. Therefore, we invite you to submit a revised version of the manuscript that addresses the points raised during the review process.

Please submit your revised manuscript by Apr 01 2026 11:59PM. If you will need more time than this to complete your revisions, please reply to this message or contact the journal office at plosone@plos.org. When you're ready to submit your revision, log on to https://www.editorialmanager.com/pone/ and select the 'Submissions Needing Revision' folder to locate your manuscript file.

- A letter that responds to each point raised by the academic editor and reviewer(s). You should upload this letter as a separate file labeled 'Response to Reviewers'.
- A marked-up copy of your manuscript that highlights changes made to the original version. You should upload this as a separate file labeled 'Revised Manuscript with Track Changes'.
- An unmarked version of your revised paper without tracked changes. You should upload this as a separate file labeled 'Manuscript'.

If you would like to make changes to your financial disclosure, please include your updated statement in your cover letter. Guidelines for resubmitting your figure files are available below the reviewer comments at the end of this letter.

If applicable, we recommend that you deposit your laboratory protocols in protocols.io to enhance the reproducibility of your results. Protocols.io assigns your protocol its own identifier (DOI) so that it can be cited independently in the future. For instructions see: https://journals.plos.org/plosone/s/submission-guidelines#loc-laboratory-protocols. Additionally, PLOS ONE offers an option for publishing peer-reviewed Lab Protocol articles, which describe protocols hosted on protocols.io. Read more information on sharing protocols at https://plos.org/protocols?utm_medium=editorial-email&utm_source=authorletters&utm_campaign=protocols.

We look forward to receiving your revised manuscript.

Kind regards,

Agnese Sbrollini

Academic Editor

PLOS One

Journal Requirements:

When submitting your revision, we need you to address these additional requirements.

1.Please ensure that your manuscript meets PLOS ONE's style requirements, including those for file naming. The PLOS ONE style templates can be found at

https://journals.plos.org/plosone/s/file?id=wjVg/PLOSOne_formatting_sample_main_body.pdf and

https://journals.plos.org/plosone/s/file?id=ba62/PLOSOne_formatting_sample_title_authors_affiliations.pdf

2. Thank you for stating the following in the Competing Interests/Financial Disclosure * (delete as necessary) section:

“I have read the journal's policy and the authors of this manuscript have the following competing interests. Melody International, Ltd. (Takamatsu, Japan), loaned the iCTG devices to the study group free of charge.”

We note that you received funding from a commercial source: “Melody International, Ltd.”

Please provide an amended Competing Interests Statement that explicitly states this commercial funder, along with any other relevant declarations relating to employment, consultancy, patents, products in development, marketed products, etc.

Within this Competing Interests Statement, please confirm that this does not alter your adherence to all PLOS ONE policies on sharing data and materials by including the following statement: "This does not alter our adherence to PLOS ONE policies on sharing data and materials.” (as detailed online in our guide for authors http://journals.plos.org/plosone/s/competing-interests).  If there are restrictions on sharing of data and/or materials, please state these. Please note that we cannot proceed with consideration of your article until this information has been declared.

Please include your amended Competing Interests Statement within your cover letter. We will change the online submission form on your behalf.

3. In the online submission form, you indicated that your data is available only on request from a third party. Please note that your Data Availability Statement is currently missing [the name of the third party contact or institution / contact details for the third party, such as an email address or a link to where data requests can be made]. Please update your statement with the missing information.

4. One of the noted authors is a group or consortium “Japan Association of Obstetricians and Gynecologists Telemedicine Proof-of-Concept Research Group” In addition to naming the author group, please list the individual authors and affiliations within this group in the acknowledgments section of your manuscript. Please also indicate clearly a lead author for this group along with a contact email address.

5. Please include your full ethics statement in the ‘Methods’ section of your manuscript file. In your statement, please include the full name of the IRB or ethics committee who approved or waived your study, as well as whether or not you obtained informed written or verbal consent. If consent was waived for your study, please include this information in your statement as well.

6. Please upload a copy of Figure 1, 2, 3 and 4, to which you refer in your text on page 10, 11 and 12. If the figure is no longer to be included as part of the submission please remove all reference to it within the text.

7. We note that Figure 1 in your submission contain map images which may be copyrighted. All PLOS content is published under the Creative Commons Attribution License (CC BY 4.0), which means that the manuscript, images, and Supporting Information files will be freely available online, and any third party is permitted to access, download, copy, distribute, and use these materials in any way, even commercially, with proper attribution. For these reasons, we cannot publish previously copyrighted maps or satellite images created using proprietary data, such as Google software (Google Maps, Street View, and Earth). For more information, see our copyright guidelines: http://journals.plos.org/plosone/s/licenses-and-copyright.

We require you to either (1) present written permission from the copyright holder to publish these figures specifically under the CC BY 4.0 license, or (2) remove the figures from your submission:

1. You may seek permission from the original copyright holder of Figure 1 to publish the content specifically under the CC BY 4.0 license.

We recommend that you contact the original copyright holder with the Content Permission Form (http://journals.plos.org/plosone/s/file?id=7c09/content-permission-form.pdf) and the following text:

“I request permission for the open-access journal PLOS ONE to publish XXX under the Creative Commons Attribution License (CCAL) CC BY 4.0 (http://creativecommons.org/licenses/by/4.0/). Please be aware that this license allows unrestricted use and distribution, even commercially, by third parties. Please reply and provide explicit written permission to publish XXX under a CC BY license and complete the attached form.”

Please upload the completed Content Permission Form or other proof of granted permissions as an "Other" file with your submission.

In the figure caption of the copyrighted figure, please include the following text: “Reprinted from [ref] under a CC BY license, with permission from [name of publisher], original copyright [original copyright year].”

2. If you are unable to obtain permission from the original copyright holder to publish these figures under the CC BY 4.0 license or if the copyright holder’s requirements are incompatible with the CC BY 4.0 license, please either i) remove the figure or ii) supply a replacement figure that complies with the CC BY 4.0 license. Please check copyright information on all replacement figures and update the figure caption with source information. If applicable, please specify in the figure caption text when a figure is similar but not identical to the original image and is therefore for illustrative purposes only.

The following resources for replacing copyrighted map figures may be helpful:

USGS National Map Viewer (public domain): http://viewer.nationalmap.gov/viewer/

The Gateway to Astronaut Photography of Earth (public domain): http://eol.jsc.nasa.gov/sseop/clickmap/

Maps at the CIA (public domain): https://www.cia.gov/library/publications/the-world-factbook/index.html and https://www.cia.gov/library/publications/cia-maps-publications/index.html

NASA Earth Observatory (public domain): http://earthobservatory.nasa.gov/

Landsat: http://landsat.visibleearth.nasa.gov/

USGS EROS (Earth Resources Observatory and Science (EROS) Center) (public domain): http://eros.usgs.gov/#

Natural Earth (public domain): http://www.naturalearthdata.com/

8. We notice that your supplementary figures are uploaded with the file type 'Figure'. Please amend the file type to 'Supporting Information'. Please ensure that each Supporting Information file has a legend listed in the manuscript after the references list.

9. If the reviewer comments include a recommendation to cite specific previously published works, please review and evaluate these publications to determine whether they are relevant and should be cited. There is no requirement to cite these works unless the editor has indicated otherwise.

[Note: HTML markup is below. Please do not edit.]

Reviewer's Responses to Questions

**Comments to the Author**

1. Is the manuscript technically sound, and do the data support the conclusions?

Reviewer #1: Partly

Reviewer #2: Yes

**********

2. Has the statistical analysis been performed appropriately and rigorously?

Reviewer #1: N/A

Reviewer #2: I Don't Know

**********

3. Have the authors made all data underlying the findings in their manuscript fully available?

The PLOS Data policy

Reviewer #1: No

Reviewer #2: No

**********

4. Is the manuscript presented in an intelligible fashion and written in standard English?

Reviewer #1: Yes

Reviewer #2: Yes

**********

Reviewer #1: Thank you for the opportunity to review this paper.

1. I believe that the key of this manuscript is "What is new". I understood the usability of mobile CTG RM during the maternal transport has not been reported yet according to this manuscript. If so, I will find this manuscript sounds novel and would like authors to emphasize this point. I did not get "what is known" and "what is unknown" regarding this topic in the Abstract and Introduction, especially focusing on the obstetric insights. I would recommend that authors summarize them in the Abstract and the Introduction.

2. I would question if the authors can say "mobile CTG RM during emergency maternal transport is feasible" without the discussion about the cost of introduction of this system (line 50). Similarly, I would say it is questionable whether authors can say "it is at least not inferior to previous management methods" (line 251), because there is no comparison between them. I did not see the data regarding the cost and I am not sure if this can be used at very rural places and on the helicopter. I would like to get the information about what we can do if the system is broken. Moreover, I would like to read the discussion about CTG RM can be better for the management than telecommunication with the attending physician who can directly observe CTG during the maternal transport.

3. Speaking of Table 1, I was wondering if this system is useful for pregnant women <28 weeks of gestation. I was also wondering if this system is hard to attach to the pregnant women whose BMI >35 kg/m2. What is possible preterm labor? Is this different from preterm labor? I was wondering in how many cases authors observed abnormal fetal heart rate patterns. I was also wondering if the diagnosis before transport, during the transport, and after the transport are matched. I believe these parameters are important to discuss the feasibility of this device.

4. I would like to ask if ununified reasons for maternal transport and gestational ages in this analysis can be the potential limitations. I would say mobile CTG RM may not be required during the stable maternal transport like threatened preterm births just diagnosed with short cervix.

Minor comments:

I was wondering how long it would take to attach this system to the patient and it would get the signals (lines 98-100).

I would recommend that authors include URL of Google Map (line 109).

I was curious if maternal heart rate can be also monitored using this device (line 224).

The footnotes are essential to indicate what Q1, Q2, Q3, and Q4 are asking in Table 2 and 3.

Reviewer #2: I am astonished that the authors consider it important to ask the paramedics their opinion of the acceptability of using CTGs during transport but make njot mention at all regarding the views of the women themselves. I would only recommend publication if data concerning the acceptability to women is included. Neglecting to include the views of the women displays a lack of patient-centered care that is not appropriate. If this data is available, please include it. If it was not obtained then I cannot recommend publication, regardless of any other issues. I appreciate this is a proof of concept and from the point of view of technical feasibility the technique seems to have been demonstrated to work. However please revise if possible, taking the views of patients into account.

**********

what does this mean?). If published, this will include your full peer review and any attached files.

If you choose “no”, your identity will remain anonymous but your review may still be made public.

**Do you want your identity to be public for this peer review?**  For information about this choice, including consent withdrawal, please see our Privacy Policy

Reviewer #1: No

Reviewer #2: No

**********

[NOTE: If reviewer comments were submitted as an attachment file, they will be attached to this email and accessible via the submission site. Please log into your account, locate the manuscript record, and check for the action link "View Attachments". If this link does not appear, there are no attachment files.]

To ensure your figures meet our technical requirements, please review our figure guidelines: https://journals.plos.org/plosone/s/figures

You may also use PLOS’s free figure tool, NAAS, to help you prepare publication quality figures: https://journals.plos.org/plosone/s/figures#loc-tools-for-figure-preparation.

NAAS will assess whether your figures meet our technical requirements by comparing each figure against our figure specifications.

# Author comment: Author response to Decision Letter 1

**DOI:** 10.1371/journal.pone.0361118.r002

23 Mar 2026

Thank you very much for your detailed review. I have revised the manuscript based on your comments. I have also listed the revisions in the separate document titled “Responses to Reviewers” that I have attached.

Attachment Submitted filename: Response to Reviewers.docx

# Peer review: Decision Letter 1

**DOI:** 10.1371/journal.pone.0361118.r003

**Authors:** Agnese Sbrollini

21 May 2026

Dear Dr. Tamaru,

Thank you for submitting your manuscript to PLOS ONE. After careful consideration, we feel that it has merit but does not fully meet PLOS ONE’s publication criteria as it currently stands. Therefore, we invite you to submit a revised version of the manuscript that addresses the points raised during the review process.

Please submit your revised manuscript by Jul 05 2026 11:59PM. If you will need more time than this to complete your revisions, please reply to this message or contact the journal office at plosone@plos.org. When you're ready to submit your revision, log on to https://www.editorialmanager.com/pone/ and select the 'Submissions Needing Revision' folder to locate your manuscript file.

- A letter that responds to each point raised by the academic editor and reviewer(s). You should upload this letter as a separate file labeled 'Response to Reviewers'.
- A marked-up copy of your manuscript that highlights changes made to the original version. You should upload this as a separate file labeled 'Revised Manuscript with Track Changes'.
- An unmarked version of your revised paper without tracked changes. You should upload this as a separate file labeled 'Manuscript'.

If you would like to make changes to your financial disclosure, please include your updated statement in your cover letter. Guidelines for resubmitting your figure files are available below the reviewer comments at the end of this letter.

If applicable, we recommend that you deposit your laboratory protocols in protocols.io to enhance the reproducibility of your results. Protocols.io assigns your protocol its own identifier (DOI) so that it can be cited independently in the future. For instructions see: https://journals.plos.org/plosone/s/submission-guidelines#loc-laboratory-protocols. Additionally, PLOS ONE offers an option for publishing peer-reviewed Lab Protocol articles, which describe protocols hosted on protocols.io. Read more information on sharing protocols at https://plos.org/protocols?utm_medium=editorial-email&utm_source=authorletters&utm_campaign=protocols.

As the corresponding author, your ORCID iD is verified in the submission system and will appear in the published article. PLOS supports the use of ORCID, and we encourage all coauthors to register for an ORCID iD and use it as well. Please encourage your coauthors to verify their ORCID iD within the submission system before final acceptance, as unverified ORCID iDs will not appear in the published article. *Only*  the individual author can complete the verification step; PLOS staff *cannot*  verify ORCID iDs on behalf of authors.

We look forward to receiving your revised manuscript.

Kind regards,

Agnese Sbrollini

Academic Editor

PLOS One

Journal Requirements:

If the reviewer comments include a recommendation to cite specific previously published works, please review and evaluate these publications to determine whether they are relevant and should be cited. There is no requirement to cite these works unless the editor has indicated otherwise.

[Note: HTML markup is below. Please do not edit.]

Reviewers' comments:

Reviewer's Responses to Questions

**Comments to the Author**

Reviewer #1: All comments have been addressed

Reviewer #2: (No Response)

**********

2. Is the manuscript technically sound, and do the data support the conclusions?

Reviewer #1: Partly

Reviewer #2: Partly

**********

3. Has the statistical analysis been performed appropriately and rigorously?

Reviewer #1: N/A

Reviewer #2: I Don't Know

**********

4. Have the authors made all data underlying the findings in their manuscript fully available?

The PLOS Data policy

Reviewer #1: No

Reviewer #2: Yes

**********

5. Is the manuscript presented in an intelligible fashion and written in standard English?

Reviewer #1: Yes

Reviewer #2: Yes

**********

Reviewer #1: I believe that all comments have been properly addressed. However, some English expressions still sound unnatural. I recommend that the authors have the manuscript reviewed by a native English speaker.

Reviewer #2: Thank you for your comment concerning the views of the patients. I appreciate that you did not survey the patients at the time of the survey, meaning that you have no data available concerning patient acceptability. You have justified this by stating that when patients used the monitoring device themselves at home they found it acceptable and this implies that therefore they would also find it acceptable during transport. However, I consider that these are two very different circumstances and acceptability in one cannot be taken to mean that use is necessarily acceptable in the other. I really cannot recommend this paper for publication unless it includes a reflection of the acceptability to the patients in the ciurcumstance of transport. I hope that you will reflect on this view going forward as the views of the patients should always be considered important.

**********

what does this mean?). If published, this will include your full peer review and any attached files.

If you choose “no”, your identity will remain anonymous but your review may still be made public.

**Do you want your identity to be public for this peer review?**  For information about this choice, including consent withdrawal, please see our Privacy Policy

Reviewer #1: No

Reviewer #2: No

**********

[NOTE: If reviewer comments were submitted as an attachment file, they will be attached to this email and accessible via the submission site. Please log into your account, locate the manuscript record, and check for the action link "View Attachments". If this link does not appear, there are no attachment files.]

To ensure your figures meet our technical requirements, please review our figure guidelines: https://journals.plos.org/plosone/s/figures

You may also use PLOS’s free figure tool, NAAS, to help you prepare publication quality figures: https://journals.plos.org/plosone/s/figures#loc-tools-for-figure-preparation.

NAAS will assess whether your figures meet our technical requirements by comparing each figure against our figure specifications.

# Author comment: Author response to Decision Letter 2

**DOI:** 10.1371/journal.pone.0361118.r004

10 Jun 2026

Point-by-point responses to the reviewer:

Review Comments to the Author

Reviewer #1: I believe that all comments have been properly addressed. However, some English expressions still sound unnatural. I recommend that the authors have the manuscript reviewed by a native English speaker.

Response:

Thank you very much for your valuable comments. As with the submission of the first draft, we asked a native English-speaking scientist to review the manuscript and help us refine it for this third submission of the revised version. The revised sections are highlighted.

Reviewer #2: Thank you for your comment concerning the views of the patients. I appreciate that you did not survey the patients at the time of the survey, meaning that you have no data available concerning patient acceptability. You have justified this by stating that when patients used the monitoring device themselves at home they found it acceptable and this implies that therefore they would also find it acceptable during transport. However, I consider that these are two very different circumstances and acceptability in one cannot be taken to mean that use is necessarily acceptable in the other. I really cannot recommend this paper for publication unless it includes a reflection of the acceptability to the patients in the ciurcumstance of transport. I hope that you will reflect on this view going forward as the views of the patients should always be considered important.

Response:

Thank you very much for your valuable comments. As the reviewer pointed out, this study did not investigate the extent to which study participants accepted the use of fetal heart rate monitors during emergency transport. We have noted this as a limitation of the study in the Discussion section. In future research, we will carefully consider the point raised by the reviewer that our studies should be conducted from the perspective of the participants.

Attachment Submitted filename: Response to Reviewers R2.docx

# Peer review: Decision Letter 2

**DOI:** 10.1371/journal.pone.0361118.r005

**Authors:** Agnese Sbrollini

14 Jul 2026

Dear Dr. Tamaru,

Thank you for submitting your manuscript to PLOS ONE. After careful consideration, we feel that it has merit but does not fully meet PLOS ONE’s publication criteria as it currently stands. Therefore, we invite you to submit a revised version of the manuscript that addresses the points raised during the review process.

Please submit your revised manuscript by Aug 28 2026 11:59PM. If you will need more time than this to complete your revisions, please reply to this message or contact the journal office at plosone@plos.org. When you're ready to submit your revision, log on to https://www.editorialmanager.com/pone/ and select the 'Submissions Needing Revision' folder to locate your manuscript file.

- A letter that responds to each point raised by the academic editor and reviewer(s). You should upload this letter as a separate file labeled 'Response to Reviewers'.
- A marked-up copy of your manuscript that highlights changes made to the original version. You should upload this as a separate file labeled 'Revised Manuscript with Track Changes'.
- An unmarked version of your revised paper without tracked changes. You should upload this as a separate file labeled 'Manuscript'.

If you would like to make changes to your financial disclosure, please include your updated statement in your cover letter. Guidelines for resubmitting your figure files are available below the reviewer comments at the end of this letter.

If applicable, we recommend that you deposit your laboratory protocols in protocols.io to enhance the reproducibility of your results. Protocols.io assigns your protocol its own identifier (DOI) so that it can be cited independently in the future. For instructions see: https://journals.plos.org/plosone/s/submission-guidelines#loc-laboratory-protocols. Additionally, PLOS ONE offers an option for publishing peer-reviewed Lab Protocol articles, which describe protocols hosted on protocols.io. Read more information on sharing protocols at https://plos.org/protocols?utm_medium=editorial-email&utm_source=authorletters&utm_campaign=protocols.

As the corresponding author, your ORCID iD is verified in the submission system and will appear in the published article. PLOS supports the use of ORCID, and we encourage all coauthors to register for an ORCID iD and use it as well. Please encourage your coauthors to verify their ORCID iD within the submission system before final acceptance, as unverified ORCID iDs will not appear in the published article. *Only*  the individual author can complete the verification step; PLOS staff *cannot*  verify ORCID iDs on behalf of authors.

We look forward to receiving your revised manuscript.

Kind regards,

Agnese Sbrollini

Academic Editor

PLOS One

Journal Requirements:

If the reviewer comments include a recommendation to cite specific previously published works, please review and evaluate these publications to determine whether they are relevant and should be cited. There is no requirement to cite these works unless the editor has indicated otherwise.

[Note: HTML markup is below. Please do not edit.]

Reviewers' comments:

Reviewer's Responses to Questions

**Comments to the Author**

Reviewer #1: All comments have been addressed

Reviewer #3: (No Response)

**********

2. Is the manuscript technically sound, and do the data support the conclusions?

Reviewer #1: (No Response)

Reviewer #3: Partly

**********

3. Has the statistical analysis been performed appropriately and rigorously?

Reviewer #1: (No Response)

Reviewer #3: Yes

**********

4. Have the authors made all data underlying the findings in their manuscript fully available?

The PLOS Data policy

Reviewer #1: (No Response)

Reviewer #3: Yes

**********

5. Is the manuscript presented in an intelligible fashion and written in standard English?

Reviewer #1: (No Response)

Reviewer #3: Yes

**********

Reviewer #1: I believe that all comments have been properly addressed and the current manuscript is written in standard English.

Reviewer #3: This study demonstrates clear feasibility and clinical value for mobile CTG during ambulance transport. Methodology is sound, questionnaire data strengthens acceptance findings, and the manuscript is well-written. I recommend minor revisions regarding generalizability and suggest clarifying how connectivity issues were managed during longer transports.

**********

what does this mean?). If published, this will include your full peer review and any attached files.

If you choose “no”, your identity will remain anonymous but your review may still be made public.

**Do you want your identity to be public for this peer review?**  For information about this choice, including consent withdrawal, please see our Privacy Policy

Reviewer #1: No

Reviewer #3: **Yes:**  Prerna Mehta

**********

[NOTE: If reviewer comments were submitted as an attachment file, they will be attached to this email and accessible via the submission site. Please log into your account, locate the manuscript record, and check for the action link "View Attachments". If this link does not appear, there are no attachment files.]

To ensure your figures meet our technical requirements, please review our figure guidelines: https://journals.plos.org/plosone/s/figures

You may also use PLOS’s free figure tool, NAAS, to help you prepare publication quality figures: https://journals.plos.org/plosone/s/figures#loc-tools-for-figure-preparation.

NAAS will assess whether your figures meet our technical requirements by comparing each figure against our figure specifications.

# Author comment: Author response to Decision Letter 3

**DOI:** 10.1371/journal.pone.0361118.r006

16 Jul 2026

Point-by-point responses to the reviewer:

Review Comments to the Author

Reviewer #1: I believe that all comments have been properly addressed and the current manuscript is written in standard English.

Response:

Thank you for confirming that all comments have been appropriately addressed and that the manuscript is written in standard English.

Reviewer #3: This study demonstrates clear feasibility and clinical value for mobile CTG during ambulance transport. Methodology is sound, questionnaire data strengthens acceptance findings, and the manuscript is well-written. I recommend minor revisions regarding generalizability and suggest clarifying how connectivity issues were managed during longer transports.

Response:

Thank you very much for your valuable comments. We added a statement to the Discussion addressing the generalizability of our findings in terms of the study setting, participating facilities, transport distance, connectivity, and FHR categories. We also clarified the procedures for managing connectivity issues, including during longer transports, in the Methods and noted in the Results that no connectivity-related problems occurred during the study.

Attachment Submitted filename: Response to Reviewers R3.docx

# Peer review: Decision Letter 3

**DOI:** 10.1371/journal.pone.0361118.r007

**Authors:** Agnese Sbrollini

28 Sep 2026

Dear Dr. Tamaru,

Thank you for submitting your manuscript to PLOS One. After careful consideration, we feel that it has merit but does not fully meet PLOS One’s publication criteria as it currently stands. Therefore, we invite you to submit a revised version of the manuscript that addresses the points raised during the review process.

Please submit your revised manuscript by Nov 12 2026 11:59PM. If you will need more time than this to complete your revisions, please reply to this message or contact the journal office at plosone@plos.org. When you're ready to submit your revision, log on to https://www.editorialmanager.com/pone/ and select the 'Submissions Needing Revision' folder to locate your manuscript file.

- A letter that responds to each point raised by the academic editor and reviewer(s). You should upload this letter as a separate file labeled 'Response to Reviewers'.
- A marked-up copy of your manuscript that highlights changes made to the original version. You should upload this as a separate file labeled 'Revised Manuscript with Track Changes'.
- An unmarked version of your revised paper without tracked changes. You should upload this as a separate file labeled 'Manuscript'.

If you would like to make changes to your financial disclosure, please include your updated statement in your cover letter. Guidelines for resubmitting your figure files are available below the reviewer comments at the end of this letter.

If applicable, we recommend that you deposit your laboratory protocols in protocols.io to enhance the reproducibility of your results. Protocols.io assigns your protocol its own identifier (DOI) so that it can be cited independently in the future. For instructions see: https://journals.plos.org/plosone/s/submission-guidelines#loc-laboratory-protocols. Additionally, PLOS One offers an option for publishing peer-reviewed Lab Protocol articles, which describe protocols hosted on protocols.io. Read more information on sharing protocols at https://plos.org/protocols?utm_medium=editorial-email&utm_source=authorletters&utm_campaign=protocols.

As the corresponding author, your ORCID iD is verified in the submission system and will appear in the published article. PLOS supports the use of ORCID, and we encourage all coauthors to register for an ORCID iD and use it as well. Please encourage your coauthors to verify their ORCID iD within the submission system before final acceptance, as unverified ORCID iDs will not appear in the published article. *Only*  the individual author can complete the verification step; PLOS staff *cannot*  verify ORCID iDs on behalf of authors.

We look forward to receiving your revised manuscript.

Kind regards,

Agnese Sbrollini

Academic Editor

PLOS One

Journal Requirements:

If the reviewer comments include a recommendation to cite specific previously published works, please review and evaluate these publications to determine whether they are relevant and should be cited. There is no requirement to cite these works unless the editor has indicated otherwise.

[Note: HTML markup is below. Please do not edit.]

Reviewers' comments:

Reviewer's Responses to Questions

**Comments to the Author**

Reviewer #1: All comments have been addressed

Reviewer #3: All comments have been addressed

**********

2. Is the manuscript technically sound, and do the data support the conclusions?

Reviewer #1: (No Response)

Reviewer #3: Partly

**********

3. Has the statistical analysis been performed appropriately and rigorously?

Reviewer #1: (No Response)

Reviewer #3: Yes

**********

4. Have the authors made all data underlying the findings in their manuscript fully available?

The PLOS Data policy

Reviewer #1: (No Response)

Reviewer #3: Yes

**********

5. Is the manuscript presented in an intelligible fashion and written in standard English?

Reviewer #1: (No Response)

Reviewer #3: Yes

**********

Reviewer #1: I confirmed that all of my comments had been appropriately addressed in the previous round of review. I believe that the manuscript has been substantially improved, particularly by the addition of a statement addressing the generalizability of the findings with respect to the study setting, participating facilities, transport distance, connectivity, and FHR categories.

Reviewer #3: The abstract provides a clear rationale for evaluating mobile cardiotocograph (CTG) devices during emergency maternal transport, but it lacks critical structure, results, and technical specifications regarding sample size and equipment, alongside a typographical error in the keywords ("moblie"). The text terminates prematurely without providing data transmission success rates or statistical outcomes, which are essential for a standard PLOS ONE submission.

**********

what does this mean?). If published, this will include your full peer review and any attached files.

If you choose “no”, your identity will remain anonymous but your review may still be made public.

**Do you want your identity to be public for this peer review?**  For information about this choice, including consent withdrawal, please see our Privacy Policy

Reviewer #1: No

Reviewer #3: **Yes:**  Prerna Mehta

**********

[NOTE: If reviewer comments were submitted as an attachment file, they will be attached to this email and accessible via the submission site. Please log into your account, locate the manuscript record, and check for the action link "View Attachments". If this link does not appear, there are no attachment files.]

To ensure your figures meet our technical requirements, please review our figure guidelines: https://journals.plos.org/plosone/s/figures

You may also use PLOS’s free figure tool, NAAS, to help you prepare publication quality figures: https://journals.plos.org/plosone/s/figures#loc-tools-for-figure-preparation.

NAAS will assess whether your figures meet our technical requirements by comparing each figure against our figure specifications.

# Author comment: Author response to Decision Letter 4

**DOI:** 10.1371/journal.pone.0361118.r008

1 Oct 2026

In response to the editorial office’s Data Availability request, we have provided the anonymized minimal dataset as a Supporting Information file (S1 Data) and updated the Data Availability Statement accordingly. Institutional permission for public release of the anonymized data has been confirmed. We also corrected an error in the reported median transport time during data reconciliation. Our responses to the reviewer comments are provided in the attached response document.

Attachment Submitted filename: PONE-D-25-57871R4_Response_to_Reviewers_Editorial_Office.docx

# Peer review: Decision Letter 4

**DOI:** 10.1371/journal.pone.0361118.r009

**Authors:** Fumihiko Namba

7 Oct 2026

Feasibility of using a mobile cardiotocograph device in an ambulance during maternal transport

PONE-D-25-57871R4

Dear Dr. Tamaru,

We’re pleased to inform you that your manuscript has been judged scientifically suitable for publication and will be formally accepted for publication once it meets all outstanding technical requirements.

Within one week, you’ll receive an e-mail detailing the required amendments. When these have been addressed, you’ll receive a formal acceptance letter and your manuscript will be scheduled for publication.

An invoice will be generated when your article is formally accepted. Please note, if your institution has a publishing partnership with PLOS and your article meets the relevant criteria, all or part of your publication costs will be covered. Please make sure your user information is up-to-date by logging into Editorial Manager at Editorial Manager® and clicking the ‘Update My Information' link at the top of the page. For questions related to billing, please contact billing support.

If your institution or institutions have a press office, please notify them about your upcoming paper to help maximize its impact. If they’ll be preparing press materials, please inform our press team as soon as possible -- no later than 48 hours after receiving the formal acceptance. Your manuscript will remain under strict press embargo until 2 pm Eastern Time on the date of publication. For more information, please contact onepress@plos.org.

Kind regards,

Fumihiko Namba

Academic Editor

PLOS One

Additional Editor Comments (optional):

Reviewers' comments:

Reviewer's Responses to Questions

**Comments to the Author**

Reviewer #3: All comments have been addressed

**********

2. Is the manuscript technically sound, and do the data support the conclusions?

Reviewer #3: Yes

**********

3. Has the statistical analysis been performed appropriately and rigorously?

Reviewer #3: Yes

**********

4. Have the authors made all data underlying the findings in their manuscript fully available?

The PLOS Data policy

Reviewer #3: Yes

**********

5. Is the manuscript presented in an intelligible fashion and written in standard English?

Reviewer #3: Yes

**********

Reviewer #3: This manuscript has been significantly improved in this revision and fully addresses the remaining queries raised by the reviewers and the editorial office. The authors have successfully resolved the typographical error in the keywords and integrated crucial quantitative details into the abstract, such as the participant sample size, specific mobile device deployment, and detailed connectivity outcomes. Crucially, they verified their source records to fix the typographical error in the median transport time, changing it to 34.5 minutes. Furthermore, they have fully complied with the journal’s data policy by providing a completely anonymized minimal dataset (S1 Data) as supporting information and updating their Data Availability Statement accordingly. All technical, ethical, and structural requirements for publication have been met, and the manuscript is now ready for formal acceptance.

**********

what does this mean?). If published, this will include your full peer review and any attached files.

If you choose “no”, your identity will remain anonymous but your review may still be made public.

**Do you want your identity to be public for this peer review?**  For information about this choice, including consent withdrawal, please see our Privacy Policy

Reviewer #3: **Yes:**  Prerna Mehta

**********

# Editor report: Acceptance letter

**DOI:** 10.1371/journal.pone.0361118.r010

**Authors:** Fumihiko Namba

PONE-D-25-57871R4

PLOS One

Dear Dr. Tamaru,

I'm pleased to inform you that your manuscript has been deemed suitable for publication in PLOS One. Congratulations! Your manuscript is now being handed over to our production team.

At this stage, our production department will prepare your paper for publication. This includes ensuring the following:

* All references, tables, and figures are properly cited

* All relevant supporting information is included in the manuscript submission,

* There are no issues that prevent the paper from being properly typeset

You will receive further instructions from the production team, including instructions on how to review your proof when it is ready. Please keep in mind that we are working through a large volume of accepted articles, so please give us a few days to review your paper and let you know the next and final steps.

Lastly, if your institution or institutions have a press office, please let them know about your upcoming paper now to help maximize its impact. If they'll be preparing press materials, please inform our press team within the next 48 hours. Your manuscript will remain under strict press embargo until 2 pm Eastern Time on the date of publication. For more information, please contact onepress@plos.org.

You will receive an invoice from PLOS for your publication fee after your manuscript has reached the completed accept phase. If you receive an email requesting payment before acceptance or for any other service, this may be a phishing scheme. Learn how to identify phishing emails and protect your accounts at https://explore.plos.org/phishing.

If we can help with anything else, please email us at customercare@plos.org.

Thank you for submitting your work to PLOS One and supporting open access.

Kind regards,

PLOS One Editorial Office Staff

on behalf of

Dr. Fumihiko Namba

Academic Editor

PLOS One
